# Optimization of energy production and central carbon metabolism in a non-respiring eukaryote

**DOI:** 10.1016/j.cub.2023.04.046

**Published:** 2023-05-09

**Authors:** Sara Alam, Ying Gu, Polina Reichert, Jürg Bähler, Snezhana Oliferenko

**Affiliations:** 1 The Francis Crick Institute, 1 Midland Road, London NW1 1AT, UK; 2 Randall Centre for Cell and Molecular Biophysics, School of Basic and Medical Biosciences, King’s College London, London SE1 1UL, UK; 3 School of Biological and Behavioural Sciences, Queen Mary University of London, Mile End Road, London E1 4NS, UK; 4 Institute of Healthy Ageing, Department of Genetics, Evolution and Environment, University College London, London WC1E 6BT, UK

## Abstract

Most eukaryotes respire oxygen, using it to generate biomass and energy. However, a few organisms have lost the capacity to respire. Understanding how they manage biomass and energy production may illuminate the critical points at which respiration feeds into central carbon metabolism and explain possible routes to its optimization. Here, we use two related fission yeasts, *Schizosaccharomyces pombe* and *Schizosaccharomyces japonicus*, as a comparative model system. We show that although *S. japonicus* does not respire oxygen, unlike *S. pombe*, it is capable of efficient NADH oxidation, amino acid synthesis, and ATP generation. We probe possible optimization strategies through the use of stable isotope tracing metabolomics, mass isotopologue distribution analysis, genetics, and physiological experiments. *S. japonicus* appears to have optimized cytosolic NADH oxidation via glycerol-3-phosphate synthesis. It runs a fully bifurcated TCA pathway, sustaining amino acid production. Finally, we propose that it has optimized glycolysis to maintain high ATP/ ADP ratio, in part by using the pentose phosphate pathway as a glycolytic shunt, reducing allosteric inhibition of glycolysis and supporting biomass generation. By comparing two related organisms with vastly different metabolic strategies, our work highlights the versatility and plasticity of central carbon metabolism in eukaryotes, illuminating critical adaptations supporting the preferential use of glycolysis over oxidative phosphorylation.

## Introduction

Establishing the rules of carbon metabolism, which produces biomass and energy, is critical for our understanding of life, from evolution to development to disease.^1–6^ In glycolysis, a molecule of glucose is catabolized to pyruvate, generating two ATP molecules. Pyruvate may be decarboxylated to acetaldehyde and then reduced to ethanol through fermentation, which oxidizes the NADH generated by glycolysis, rendering this metabolic strategy redox-neutral. Alternatively, in respiration, pyruvate may be converted to acetyl-CoA, which then enters the tricarboxylic acid (TCA) cycle. Each round of the cycle provides precursors for amino acids and nucleotides, as well as generating NADH and succinate. NADH and succinate are oxidized via the electron transport chain (ETC), generating a potential across the inner mitochondrial membrane to power ATP synthesis. In yeasts that do not have the proton-pumping ETC complex I, respiration together with the catabolism of glucose via glycolysis can generate up to 16–18 ATP per glucose.^7–11^ Most eukaryotes are capable of both respiration and fermentation, but cells may choose one metabolic strategy over the other.^12,13^ For instance, Crabtree-positive yeasts such as *Schizosaccharomyces pombe* (*S. pombe*) and *Saccharomyces cerevisiae* (*S. cerevisiae*) channel more glucose toward fermentation when ample glucose is available.^13–15^ Fermentation is less efficient at generating ATP, but it produces it quickly and at a low cost, while allowing Crabtree-positive species to divert glucose away from other organisms.^1,11,15–19^

Respiration may be the most efficient method also for NADH oxidation, which is essential to support growth.^20,21^ Indeed, the growth of non-respiring *S. cerevisiae* and *S. pombe* is improved by amino acid supplementation, suggesting that biomass production is limited when respiration is blocked.^13,22,23^ How is eukaryotic central carbon metabolism structured to overcome the limitations associated with the loss of respiration?

Unlike *S. pombe*, the related fission yeast *Schizosaccharomyces japonicus* (*S. japonicus*)^24–31^ thrives both in the presence and the absence of oxygen.^32–37^ Despite encoding most genes required for respiration, it does not produce coenzyme Q, does not grow on a non-fermentable carbon source glycerol, and does not consume oxygen during growth on glucose.^32,33,36,38^ We reasoned that understanding how *S. japonicus* manages its fully fermentative lifestyle might provide fundamental insights into the wiring of central carbon metabolism in eukaryotes.

## Results

### *S. japonicus* does not respire oxygen in various physiological situations

The *cox6* gene encodes an evolutionarily conserved subunit of the ETC complex IV, which is essential for respiration in *S. pombe*.^39^ To test possible contributions of respiration to *S. japonicus* physiology, we analyzed the growth requirements and oxygen consumption in the wild-type and *cox6*⊿ *S. japonicus* and the corresponding strains of *S. pombe*.

**Figure 1:**
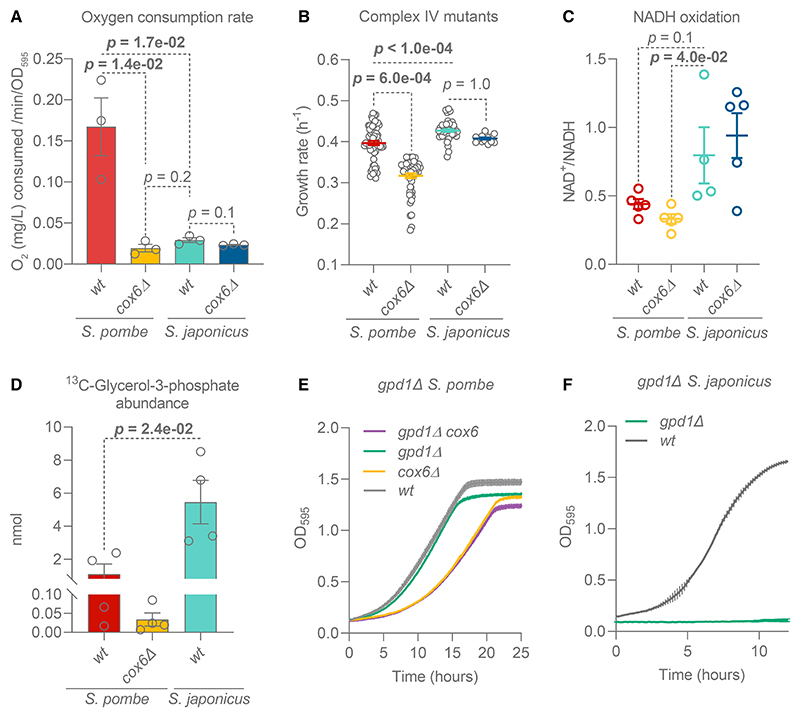
*S. japonicus* does not respire but oxidizes NADH efficiently (A) Oxygen consumption rates of *S. pombe* and *S. japonicus* wild-type (WT) and *cox6*⊿ cultures in YES medium. Means are derived from three biological replicates. (B) Growth rates of indicated strains in EMM medium. Means are derived from at least five biological repeats with three technical replicates. (C) Cellular NAD^+^/NADH ratios. Means are derived from at least four biological replicates. (D) Production of ^13^C-labeled glycerol-3-phosphate after 1 min of ^13^C_6_-glucose exposure. Means represent two biological and two technical replicates. (A–D) Error bars represent ±SEM; p values are derived from unpaired t test. (E and F) Growth curves of *S. pombe* strains (E) in EMM medium and *S. japonicus* strains (F) in YES medium. Error bars represent ±SD. Shown are the means of OD_595_ readings derived from three technical replicates, representative of three biological repeats. See also Figure S1 and Data S1A.

Consistent with previously published results,^36^ in a rich medium the wild-type *S. japonicus* exhibited a much lower oxygen consumption rate than *S. pombe* (Figure 1A). Whereas the deletion of *cox6* critically decreased oxygen consumption in *S. pombe*, we observed no such effect in *S. japonicus* (Figure 1A). This result suggested that the minimal oxygen consumption in wild-type *S. japonicus* and *cox6*⊿ *S. pombe* cells was likely due to non-respiratory oxygen-consuming processes.^40–42^ *S. japonicus* did not grow on non-fermentable carbon sources, glycerol and galactose (Figure S1A), indicating that the lack of oxygen consumption in glucose was not due to respiratory repression. We observed this behavior in several wild isolates^36,43^ and *S. japonicus var. versatilis*,^44,45^ suggesting that this feature was a species-wide trait (Figure S1B).

*S. pombe* relies on ETC for rapid growth in minimal media, where cells must synthesize most biomass precursors.^13,22^ In contrast, the deletion of *cox6* in *S. japonicus* did not impact the growth rate in the Edinburgh minimal medium (EMM) (Figure 1B). Interestingly, whereas the growth rate of *S. japonicus* in EMM was approximately 15% higher than the respiro-fermenting *S. pombe* (Figure 1B), it produced less biomass, comparable with the *S. pombe cox6*⊿ mutant (Figure S1C). *S. japonicus* grows 1.5-times faster than *S. pombe* in the rich medium, which contains many biomass precursors. *S. japonicus cox6*⊿ mutants exhibited minor attenuation in the growth rate in yeast extract and supplements (YES) medium (Figure S1D). To test whether this phenotype was due to ETC disruption, we constructed a strain lacking *rip1*, which encodes a complex III subunit essential for respiration^39^ (Figure S1A). The growth rate of *rip1*Δ *S. japonicus* cells was comparable to that of the wild-type (Figure S1D), suggesting that the minor growth phenotype of *cox6*⊿ mutants observed in the YES medium was not due to the disruption of ETC or oxidative phosphorylation. Respiration plays an important role in sporulation and mating in *S. cerevisiae* and *S. pombe*.^46,47^ However, both *cox6*⊿ and *rip1*Δ *S. japonicus* mutants showed normal mating and sporulation efficiency (Figure S1E). Furthermore, we did not observe ETC-associated defects in hyphal growth (Figure S1F). Thus, the loss of ETC components largely did not affect key physiological states of *S. japonicus*.

A key function of the ETC is NADH oxidation.^20,21^ Interestingly, *S. japonicus* exhibited a whole-cell NAD^+^/NADH ratio similar to *S. pombe*; in fact, this ratio was higher than in non-respiring *S. pombe* mutants (Figure 1C). This finding suggests that *S. japonicus* has evolved ETC-independent mechanisms to efficiently oxidize NADH.

Anaerobically grown *S. cerevisiae* depends on the reduction of dihydroxyacetone phosphate (DHAP) to glycerol-3-phosphate (G3P) by the G3P dehydrogenase Gpd1 to oxidize cytosolic NADH.^48–50^ We hypothesized that *S. japonicus* may rely on this reaction to sustain growth. We used stable isotope tracing coupled to gas chromatography mass spectrometry (GS-MS) to explore the rate of incorporation of glucose-derived carbons into the G3P pool.^51,52^ Of note, *S. japonicus* exhibited a higher rate of ^13^C incorporation into G3P as compared with *S. pombe* (Figure 1D). The non-respiring *S. pombe cox6*⊿ mutant did not upregulate G3P synthesis as compared with the wild-type. *S. japonicus* also maintained a larger pool of G3P than *S. pombe* (Figure S1G). G3P is typically used as a lipid precursor or converted to glycerol. Both intracellular and extracellular glycerol levels were comparable in *S. pombe* and *S. japonicus* (Figures S1H and S1I).

To test whether *S. japonicus* relied on DHAP reduction to sustain NADH oxidation in the absence of respiration, we generated the *gpd1*Δ strain. Strikingly, while the deletion of *gpd1* in *S. pombe* did not negatively affect its growth, regardless of respiratory activity (Figures 1E and S1J), *S. japonicus gpd1*Δ cells were virtually incapable of growth, even in the rich medium, and could not be maintained at all in the minimal medium (Figure 1F). We conclude that *S. japonicus* critically depends on Gpd1 activity, whereas *S. pombe* may have additional mechanisms to oxidize cytosolic NADH.

### *S. japonicus* operates a bifurcated TCA pathway with endogenous bicarbonate recycling, whereas *S. pombe* maintains both bifurcated and cyclic TCA variants

**Figure 2:**
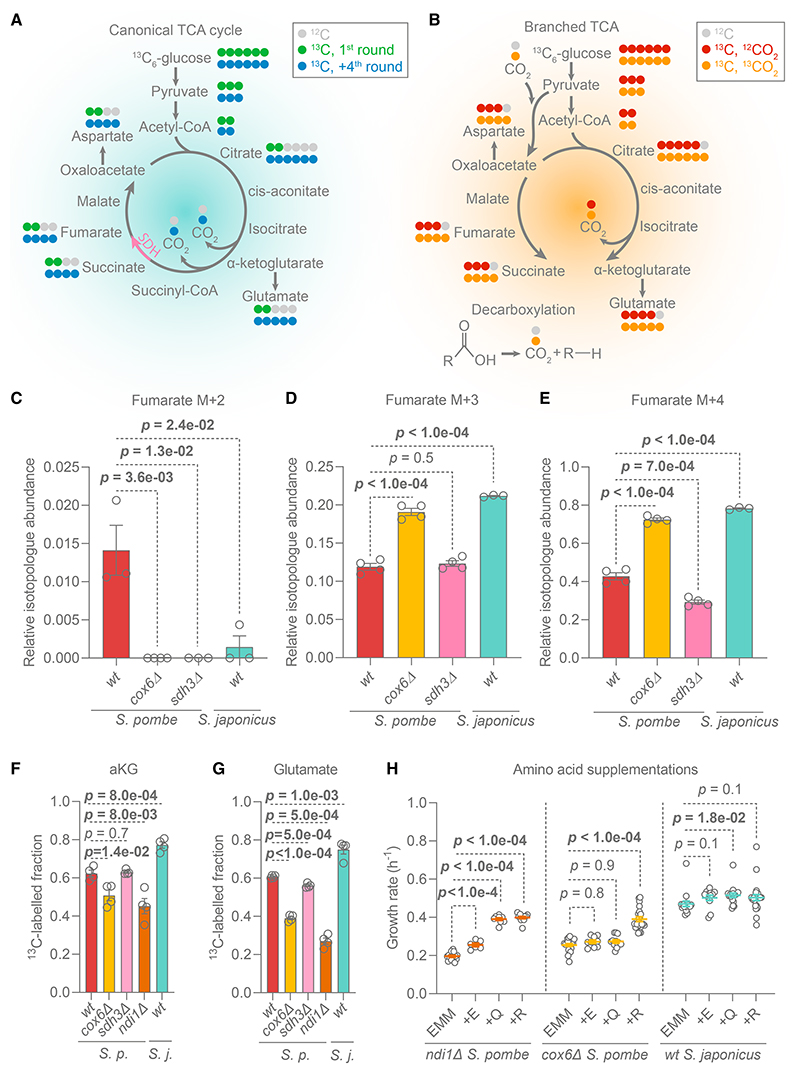
*S. japonicus* operates a bifurcated TCA pathway and efficiently synthesizes TCA-derived amino acids (A) Isotopologs of intermediates expected in the oxidative TCA cycle, after feeding ^13^C_6_-glucose. Pink arrow: the reaction catalyzed by succinate dehydrogenase (SDH). Isotopologs in green originate from the first cycle (M + 2 acetyl-CoA and M + 0 oxaloacetate). Isotopologs in blue are expected to be generated after the 4^th^ cycle.^52,57–59^ (B) Isotopologs originating from the bifurcated TCA pathway. M + 3 pyruvate may be carboxylated using M + 0 CO_2_, leading to M + 3 oxaloacetate/aspartate (red),^52,57–59^ or M + 1 CO_2_, leading to M + 4 oxaloacetate/aspartate (orange). (C–E) M + 2 (C), M + 3 (D), and M + 4 (E) fumarate fractions relative to the entire fumarate pool 30 min after ^13^C_6_-glucose addition. (F and G) ^13^C-labeled alpha-ketoglutarate (F) and glutamate (G) fractions 30 min after ^13^C_6_-glucose addition. (C–G) Shown are mean ±SEM of two biological and two technical replicates, p values were calculated using unpaired t test. (H) Growth rates of *S. pombe* and *S. japonicus* grown in EMM with 0.2 g/L of either glutamate (E), glutamine (Q), or arginine (R). Mean ±SEM of three technical and at least two biological replicates are shown, with p values generated using unpaired t test. See also Figure S2 and Data S1D.

Respiration is typically associated with the TCA cycle, which allows for the additional oxidation of glucose, enabling more ATP production.^10^ The TCA cycle also generates crucial biomass precursors, alpha-ketoglutarate (aKG) and oxaloacetate, which are used to synthesize glutamate, aspartate, and other amino acids and metabolites (Figure 2A). In anaerobic *S. cerevisiae*, the TCA cycle bifurcates, with an oxidative branch running from acetyl-CoA to aKG and a reductive branch starting from the carboxylation of pyruvate to oxaloacetate, with subsequent conversion up to succinate.^53^ In the absence of respiration, bifurcation of the TCA cycle presumably enables cells to support oxaloacetate and aKG synthesis in a redox-neutral manner (Figure 2B). We assessed the TCA cycle architecture in *S. pombe* and *S. japonicus* using stable isotope tracing metabolomics, quantifying the ratios of different TCA-intermediate isotopologs after feeding cells with ^13^C_6_-glucose (Figures 2A and 2B). To mimic the “broken” TCA cycle, we included the *S. pombe* mutant lacking Sdh3, the key subunit of the succinate dehydrogenase complex.^54–56^ We used fumarate and succinate, the product and substrate of succinate dehydrogenase, as diagnostic metabolites.

M + 2 fumarate is typically associated with the oxidative TCA cycle^52,57–59^ (Figure 2A). Indeed, only respiro-fermenting wild-type *S. pombe* showed M + 2 fumarate (Figure 2C). M + 3 fumarate likely originates from the reductive TCA branch^52,57–59^ (Figure 2B). Interestingly, both wild-type and *cox6*⊿ *S. pombe*, and wild-type *S. japonicus*, showed high M + 3 fumarate fractions (Figure 2D). This indicates that not only *S. japonicus* and non-respiring *S. pombe* but also the wild-type *S. pombe* operate the reductive TCA branch. However, the inability to respire in both fission yeasts leads to an increase in M + 3 fumarate labeling, suggesting greater use of the bifurcated TCA architecture. The oxidative branch appears to extend to succinate, as suggested by M + 2 succinate labeling in *cox6*⊿ and *sdh3*Δ *S. pombe* and wild-type *S. japonicus* (Figure S2A).

M + 4 fumarate is thought to result from repeated oxidative TCA cycles^52,57–59^ (Figure 2A). The wild-type *S. pombe* indeed showed a M + 4 fumarate signal. However, this isotopolog was also abundant in both non-respiring *cox6*⊿ *S. pombe* and the wild-type *S. japonicus* (Figure 2E). The proportion of M + 4 fumarate in *S. pombe* was only minimally affected by the deletion of *sdh3*, which disrupts the oxidative cycle.^60^ In principle, M + 4 fumarate could be produced in the reductive branch from M + 4 oxaloacetate (Figure 2B), originating from the carboxylation of the M + 3 pyruvate using M + 1 bicarbonate.^61^ Indeed, we observed M + 4 aspartate (proxy for oxaloacetate) in both fission yeasts and their TCA cycle mutants (Figure S2B). The labeled bicarbonate is likely released from decarboxylating reactions,^61^ such as conversion of pyruvate to acetaldehyde in fermentation.^10^ Thus, a large fraction of M + 4 fumarate—even in respiring cells—could be a product of the reductive TCA branch and the recycling of endogenous bicarbonate.

Interestingly, both wild-type and *sdh3*Δ *S. pombe* cells had M + 4 succinate but lacked M + 3 fractions, in contrast with *S. japonicus* and the non-respiring *cox6*⊿ *S. pombe* mutant (Figures S2C and S2D). This suggests that the reduction of fumarate to succinate by fumarate reductase in *S. pombe* favors M + 4 fumarate, possibly due to distinct compartmentalization of fumarate isotopologs in respiro-fermenting cells.

Taken together, our genetic and metabolomics data suggest that (1) *S. japonicus* operates a bifurcated TCA pathway with endogenous bicarbonate recycling and (2) the respiro-fermenting *S. pombe* uses a combination of bifurcated and cyclic TCA reactions.

### Efficient NADH oxidation, but not cyclic TCA activity, is required to sustain glutamate synthesis

It has been postulated that sufficient production of aKG-derived amino acids, such as glutamate and arginine, requires a canonical TCA cycle.^22,23,62^ Seemingly in agreement with that hypothesis, aKG and glutamate labeling from ^13^C-glucose was lower in the non-respiring *S. pombe cox6*⊿ mutant as compared with the wild-type (Figures 2F and 2G). However, both aKG and glutamate were labeled to a higher degree in *S. japonicus* than in *S. pombe* (Figures 2F and 2G). The *S. pombe sdh3*Δ mutants, which have a broken TCA cycle, labeled aKG and glutamate similarly to the wild-type and grew normally in the minimal medium (Figures 2F, 2G, and S2E). These results suggest that some aspect of respiration, rather than running the canonical TCA cycle, is important for glutamate synthesis in *S. pombe*, whereas *S. japonicus* has evolved a respiration-independent strategy to sustain amino acid production.

The oxidative branch of the TCA cycle generates NADH, which must be re-oxidized to support TCA reactions and biomass production. We wondered whether decreased NADH re-oxidation in mitochondria was responsible for the reduced aKG and glutamate labeling in *S. pombe cox6*⊿ mutants. Ndi1 is the matrix-facing NADH dehydrogenase associated with the ETC. Deleting *ndi1* did not abolish respiration in *S. pombe*, as despite the attenuation of growth rate in EMM (Figure S2E), *ndi1*Δ mutants could grow on a non-fermentable medium (Figure S2F). Interestingly, *S. pombe ndi1*Δ cells showed a pronounced reduction in aKG and glutamate labeling (Figures 2F and 2G). Accordingly, supplementation with aKG-derived amino acids—glutamate, glutamine, or arginine—improved their growth in EMM (Figure 2H). Surprisingly, the growth defect of *S. pombe cox6*⊿ mutants in EMM could be rescued by arginine but not glutamate or glutamine (Figure 2H). This finding indicates that the arginine dependency of non-respiring *cox6*⊿ *S. pombe* is not due to limited aKG production. *S. japonicus* appears to have overcome this limitation (Figure 2H).

Taken together, our results suggest that as long as cells can efficiently re-oxidize NADH generated by the oxidative TCA branch, a bifurcated TCA architecture can sustain amino acid synthesis and biomass production.

### *S. japonicus* sustains higher glycolytic activity than

#### S. pombe

**Figure 3:**
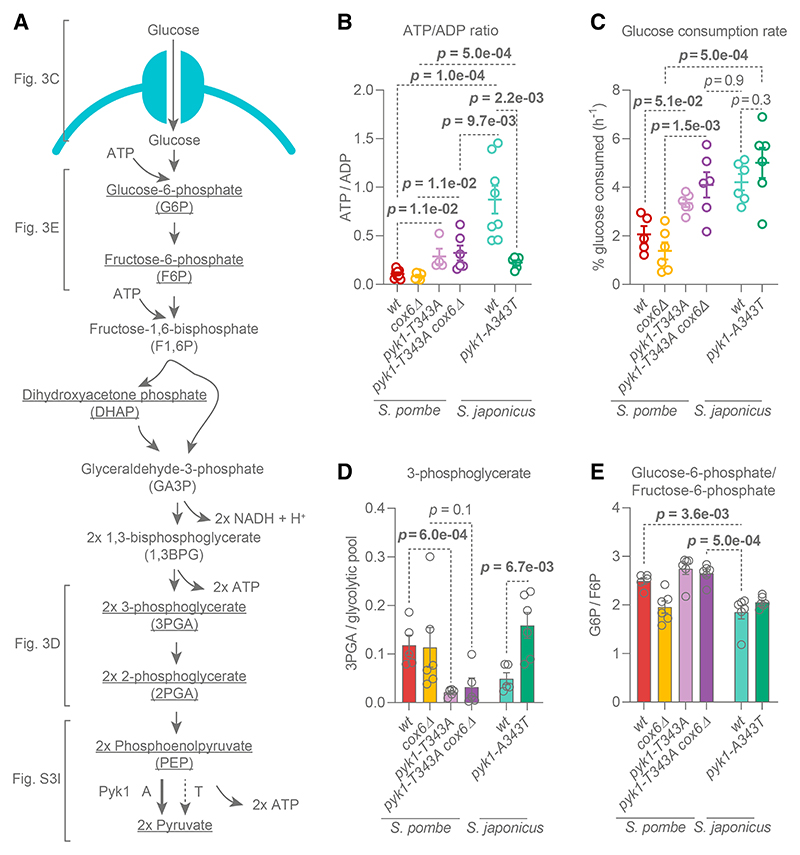
*S. japonicus* maintains higher glycolytic activity than *S. pombe* (A) Glycolysis and its outputs. Blue: plasma membrane hexose transporter. Underlined: metabolites quantified in this study. Pyruvate kinase Pyk1 is indicated, with A and T denoting the point mutation at site 343. (B) Whole-cell ATP/ADP ratios. Mean ±SEM values of at least four biological replicates. p values were calculated using unpaired t test. (C) Rate of glucose uptake in EMM. Mean ± SEM of at least five biological replicates are shown. p values were calculated using unpaired t test. (D)3-phosphoglycerate abundance relative to the sum of detected glycolytic intermediates (G6P, F6P, DHAP, 3PGA, 2PGA, PEP, and pyruvate). (E) Glucose-6-phosphate levels relative to fructose-6-phosphate. (D and E) Shown are mean ±SEM of three technical and two biological replicates. p values were calculated using unpaired t test. See also Figure S3 and Data S1E.

Respiration allows for additional oxidation of carbon substrates, producing more ATP than glycolysis alone (Figure 3A). Surprisingly, *S. japonicus* exhibited higher ATP levels (Figure S3A) and a higher ATP/ADP ratio as compared with both respiro-fermenting (wild-type) and solely fermenting (*cox6*D) *S. pombe* (Figure 3B). This suggests that the energetic output of glycolysis in *S. japonicus* is higher than that of *S. pombe*.

The laboratory “wild-type” strain of *S. pombe* has a partial loss-of-function alanine-to-threonine point mutation at position 343 (A343T) in the pyruvate kinase Pyk1, which catalyzes the last energy-yielding step of glycolysis^63^ (Figure 3A). The Pyk1 kinase in *S. japonicus* does not have this substitution. To test whether higher Pyk1 activity is solely responsible for more efficient ATP production via glycolysis in this organism, we constructed a *pyk1-A343T S. japonicus* mutant. To specifically home in on the energetic output of glycolysis, we also made a non-respiring *S. pombe* strain with higher Pyk1 activity (*pyk1-T343A cox6*D). Consistent with previous work,^63^ the higher Pyk1 activity in *S. pombe* increased the cellular ATP/ADP ratio, whereas the lower Pyk1 activity in *S. japonicus* reduced it (Figure 3B). Interestingly, the higher Pyk1 activity did not affect the total ATP levels in *S. pombe*, although the lower Pyk1 activity did reduce this pool in *S. japonicus* (Figure S3A). The ATP/ADP ratio of wild-type *S. japonicus* was still higher than that of *pyk1-T343A cox6*⊿ *S. pombe* (Figure 3B). This result is notable because both the wild-type *S. japonicus* and *pyk1-T343A cox6*⊿ *S. pombe* mutant do not respire and presumably have comparable levels of pyruvate kinase activity.

We measured the glucose uptake of exponentially growing cultures to estimate glycolytic rates in the two sister species.^64,65^ The higher activity of Pyk1 improved glucose uptake in *S. pombe*, regardless of respiratory activity (Figure 3C). The glucose uptake rate of *S. japonicus* was similar to that of the non-respiring *S. pombe* with high Pyk1 activity (*pyk1-T343A cox6*D). Interestingly, glucose uptake remained high in *S. japonicus*, even when we introduced the *S. pombe*-specific partial loss-of-function *pyk1-A343T* allele (Figure 3C). This suggests that, unlike in *S. pombe*, glycolysis in *S. japonicus* is not regulated as tightly by the pyruvate kinase. Notably, whereas the *pyk1* alleles do play a major role in modulating ATP production via glycolysis, *S. japonicus* might have evolved additional means of maximizing glycolytic output.

To further probe the regulation of glycolysis, we quantified glycolytic intermediates using GC-MS. The abundance of each intermediate was expressed as a fraction of the whole glycolytic intermediate pool, as this allowed us to pinpoint potential regulatory points (Figures S3B–S3H). Corroborating published data, we observed a release in the phosphoenolpyruvate-to-pyruvate bottleneck when introducing a more active *pyk1* allele to *S. pombe* (Figure S3I). Suggesting that the pyruvate kinase activity could indeed regulate glycolysis as a whole, we have observed a Pyk1-activity-dependent bottleneck at the 3-phosphoglycerate level in *S. pombe* (Figure 3D). In line with the predicted higher pyruvate kinase activity in *S. japonicus*, we detected the accumulation of both phosphoenolpyruvate and 3-phosphoglycerate after introducing the *S. pombe*-like *pyk1-A343T* allele to this species (Figures 3D and S3I).

Interestingly, regardless of the *pyk1* allele, *S. japonicus* exhibited a lower glucose-6-phosphate to fructose-6-phosphate (G6P/F6P) ratio than *S. pombe* (Figure 3E). This suggested that G6P was consumed more rapidly in *S. japonicus*, highlighting potential differences in upper glycolysis between the two organisms.

### *S. japonicus* may upregulate upper glycolysis via the pentose phosphate pathway

**Figure 4:**
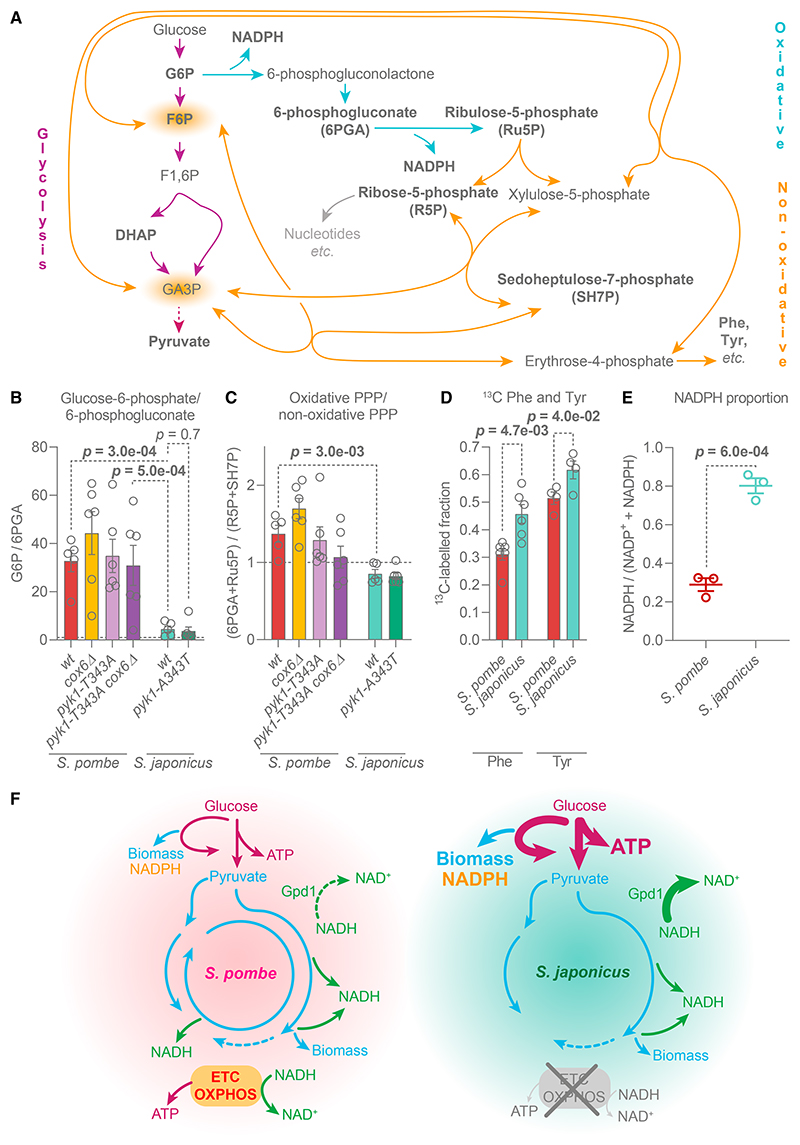
*S. japonicus* may upregulate the entry into the pentose phosphate pathway (A) PPP and its intersection with glycolysis. Bold: metabolites quantified in this study. G6P, glucose-6-phosphate; F6P, fructose-6-phosphate; F1,6BP, fructose-1,6-bisphosphate; DHAP, dihydroxyacetone phosphate; Phe, phenylalanine; Tyr, tyrosine. (B) Glucose-6-phosphate abundance normalized to 6-phosphogluconate. (C) Sum of oxidative PPP intermediates (6PGA and Ru5P) relative to non-oxidative PPP intermediates (R5P and SH7P). (B and C) Dotted lines indicate the ratio of 1. (D)^13^C-labeled phenylalanine and tyrosine fractions 10 min after ^13^C_6_-labeled glucose addition. (B–D) Mean ±SEM of two biological and two to three technical replicates. Statistical analyses were performed using unpaired t test. (E) Cellular NADPH relative to a total NADP(H) pool. Mean ±SEM of three biological replicates, p values estimated using unpaired t test. (F) A diagram summarizing the findings of this study. ETC, electron transport chain; OXPHOS, oxidative phosphorylation. See also Figure S4 and Data S1E–S1G.

A major route utilizing G6P is the pentose phosphate pathway (PPP) (Figure 4A). Interestingly, the G6P to 6-phosphogluconate (G6P/6PGA) ratio was considerably lower in *S. japonicus* as compared with *S. pombe* (Figure 4B). This suggested that *S. pombe* has a bottleneck at the PPP entry point but *S. japonicus* may channel large amounts of G6P into the PPP. This phenomenon was independent of the pyruvate kinase activity (Figure 4B). Indicating a steady-state conversion of 6PGA to ribulose-5-phosphate, the ratio of these metabolites in *S. japonicus* was close to 1 (Figure S4A).

The PPP is composed of an oxidative followed by a non-oxidative branch. The latter allows cells to redirect carbon back into glycolysis^66–70^ (Figure 4A). Interestingly, the ratio of oxidative relative to non-oxidative PPP products was higher in *S. pombe* as compared with *S. japonicus*, indicating a potential bottleneck at the transition between the two branches in the former (Figure 4C; see individual data for ribulose-5-phosphate/ribose-5-phosphate and ribose-5-phosphate/sedoheptulose-7-phosphate ratios in Figures S4B and S4C). Importantly, we detected higher labeling of phenylalanine and tyrosine, amino acids produced from the non-oxidative PPP (Figure 4A), from ^13^C-glucose in *S. japonicus* than *S. pombe* (Figure 4D).

Finally, we observed a considerably higher NADPH/total NADP(H) ratio in *S. japonicus* as compared with *S. pombe* (Figure 4E). As NADPH is a product of oxidative PPP (Figure 4A), our results indicate that this pathway may operate at a higher capacity in *S. japonicus*.

Taken together, these results indicate that *S. japonicus* may use PPP to upregulate glycolysis by preventing the accumulation of upper glycolytic intermediates, thus reducing feedback-inhibition of this pathway. Additionally, upregulated PPP could support anabolism by providing biomass precursors and NADPH.

## Discussion

Our work suggests that the non-respiring *S. japonicus* has optimized its energy and redox metabolism to support rapid growth at the expense of biomass production yield (Figure 4F).

First, *S. japonicus* relies on cytosolic DHAP reduction to reoxidize NADH (Figures 1D and 1F). Although diverting DHAP away from glycolysis may reduce ATP yield, there are several advantages to this strategy. In addition to supporting amino acid and nucleotide anabolism by regenerating NAD^+^, the high activity of G3P dehydrogenase Gpd1 may provide more glycerol backbones for lipid synthesis.^71^ Furthermore, high Gpd1 activity could prevent the build-up of DHAP, which can be converted to the toxic by-product, methylglyoxal.^72^ *S. pombe* appears to use alternative NADH oxidation strategies during non-respiratory growth (Figure 1E). Obvious candidates include alcohol dehydrogenases and the NAD(H)-dependent malic enzyme Mae2,^63,73,74^ or the lactate dehydrogenase SPAC186.08c^75^ that is absent in the genomes of either *S. japonicus* or budding yeast.^35,76,77^

Second, *S. japonicus* supports the production of amino acids derived from the TCA cycle by running a bifurcated version of this pathway. Interestingly, even *S. pombe*, which requires respiration for optimal growth, runs both bifurcated and canonical TCA cycles (Figure 2). Presumably, the bifurcated version allows for better biomass production by committing oxaloacetate and aKG to amino acid synthesis. In *S. pombe*, the bifurcated TCA pathway yielding biomass still depends on ETC-dependent NADH oxidation (Figures 2F–2H). By utilizing the bifurcated TCA pathway alongside respiration, this organism maintains rapid growth and biomass production. Yet, the fast growth of *S. japonicus* demonstrates that this is not the only effective strategy. Interestingly, *S. japonicus* has lost the NAD(H)-dependent isocitrate dehydrogenase (Idh1/2), while retaining the NADP(H)-dependent isocitrate dehydrogenase Idp1.^35,77^ Furthermore, it has lost one of the NAD(H)-dependent glutamate dehydrogenases, Gdh2, but kept the NADP(H)-specific glutamate dehydrogenase, Gdh1.^35,77–79^ This suggests that besides evolving efficient NADH oxidation pathway(s), *S. japonicus* may have alleviated the NADH burden by changing the cofactor dependencies of anabolic enzymes.

Third, *S. japonicus* maintains high ATP levels and a high ATP/ADP ratio by maximizing glycolysis, presumably through a combination of high pyruvate kinase activity and by diverting G6P through the PPP (Figures 3 and 4). Diverting G6P away from glycolysis and reintroducing carbon back at the glyceraldehyde-3-phosphate level may reduce allosteric inhibition of hexo-kinase and help maintain high glycolytic activity.^65,80^

Of note, the replacement of *S. japonicus pyk1* with the *S. pombe*-like allele of the pyruvate kinase did not reduce glucose uptake (Figure 3C). This suggests that *S. japonicus* upper glycolysis is less tightly regulated by lower glycolysis as compared with its sister species. Presumably, this feature allows *S. japonicus* to sustain rapid glycolysis in situations where it normally would be inhibited, such as low pH^81,82^ or low glucose levels.^83–85^ The latter could be particularly important because *S. japonicus* cannot rely on respiration as a metabolic strategy for dealing with starvation, unlike *S. pombe*.^12,13,56^ Interestingly, *S. japonicus* has lost the fructose-1,6-bisphosphatase Fbp1,^35,77^ rendering it incapable of gluconeogenesis.^86^ The lack of Fbp1 may also prevent the suppression of glycolysis in low glucose,^87^ which is arguably vital for a non-respiring organism. The lack of gluconeogenesis in *S. japonicus* may support the use of the non-oxidative part of the PPP as a one-way shunt into glycolysis and would prevent the recycling of F6P and glyceraldehyde-3-phosphate into the oxidative PPP.^67^ Overall, a combination of upregulated PPP, “good” pyruvate kinase, and other adaptations reducing negative feedback on glycolysis allows *S. japonicus* to maintain high ATP levels independent of oxidative phosphorylation.

Finally, the upregulation of the PPP may allow *S. japonicus* to rapidly produce nucleotides and some amino acids, together with NADPH, which is key for several anabolic pathways, e.g., lipid metabolism^88^ (Figures 4D and 4E). *S. japonicus* is highly sensitive to paraquat and hydrogen peroxide,^36^ despite high levels of NADPH, which is needed for the reduction of glutathione and thioredoxin.^89,90^ It is plausible that over the course of its life history, which has involved adaptation to anaerobic environments, this species has lost some capacity to manage oxidative stress.

*S. japonicus*, as a committed fermenting species, may successfully compete with other organisms by growing rapidly, sequestering glucose, and secreting ethanol and other toxic waste products.^36,43^ Critically, *S. japonicus* grows well both in the presence and absence of oxygen,^32–37^ allowing it to explore different ecological niches. Yet, the inability to respire restricts this species toa narrow range of carbon sources. Furthermore, the lack of respiration may account for its relatively low biomass yield (Figure S1C). This is consistent with the behavior of Crabtree-positive yeasts, which also grow rapidly but to a lower final biomass content.^11,15^ Such trade-offs may manifest in the wild, giving *S. japonicus* selective advantage only in the environments replete with nutrients and glucose. Indeed, *S. japonicus* grows at a considerably higher rate (1.6-times faster) in the rich medium, where many biomass precursors are available, as compared with the EMM. However, our work suggests that it has evolved a range of strategies to thrive even in nutritionally sub-optimal conditions, for instance in EMM, when it is forced to synthesize most biomass precursors.

*S. japonicus* shares a number of metabolic traits with the anaerobically growing *S. cerevisiae*, including the reliance on G3P dehydrogenase-dependent NADH oxidation and the use of the bifurcated TCA pathway.^48,53,91^ Its innovations may include the potential optimization of glycolysis through the PPP shunt and the extension of the oxidative TCA branch to succinate. In anaerobic budding yeast, succinate appears to be made from the oxidative TCA branch when glutamate is supplemented.^53^ Such an extension may allow *S. japonicus* to undergo the TCA substrate-level phosphorylation at the succinate-CoA ligase step. Finally, *S. japonicus* does not secrete more glycerol as compared with *S. pombe*, unlike *S. cerevisiae* that upregulates glycerol production in anoxia due to increased G3P synthesis and dephosphorylation.^48^ Coincidentally, *S. japonicus* cannot consume secreted glycerol (Figures S1A and S1B), unlike budding yeast.

Our study lays the groundwork for better understanding of central carbon metabolism in fission yeasts and beyond. We demonstrate the power of stable isotope tracing metabolomics, mass isotopologue distribution analyses, and genetic perturbations in illuminating the architecture of metabolic pathways in yeasts. Importantly, our work showcases a comparative biology approach to understanding metabolism.

Central carbon metabolism is woven tightly into the fabric of cellular biology. Understanding the plasticity of metabolism—both in ontogenetic and phylogenetic terms—may ultimately aid in explaining organismal ecology and the evolution of higher-level cellular features, such cell size and growth rate.

## Star⋆Methods

Detailed methods are provided in the online version of this paper and include the following:

○ KEY RESOURCES TABLE
○ RESOURCE AVAILABILITY
  - Lead contact
  - Materials availability
  - Data and code availability
○ EXPERIMENTAL MODEL AND SUBJECT DETAILS
○ METHOD DETAILS
  - Serial dilution assays
  - Hyphae formation assay
  - Sporulation efficiency assay
  - Molecular genetics
  - Oxygen consumption
  - Biomass yield coefficient determination
  - Glucose consumption
  - NAD(P)+/NAD(P)H quantification
  - ATP/ADP quantification
  - Gas chromatography-mass spectrometry metabolomics
  - Analysis of gas chromatography-mass spectrometry metabolomics data
○ STATISTICAL ANALYSES

## Star+Methods

### Key Resources Table

**Table:** 

REAGENT or RESOURCE		SOURCE	IDENTIFIER	
Chemicals, peptides, and recombinant proteins				
Methanol hypergrade for LC-MS LiChrosolv		Merck	Cat#1.06035	
Chloroform LiChrosolv		Merck	Cat#1.02444	
Acetonitrile hypergrade for LC-MS LiChrosolv		Merck	Cat#1.00029	
Water, Optima™ LC/MS Grade		Fisher Scientific	Cat#W6500	
13C labeled glucose		Cambridge Isotope Laboratories	Cat#CLM-1396-PK	
Scyllo-inositol		Sigma Aldrich	Cat#I8132	
Methoxyamine hydrochloride, for GC derivatization, LiChropur™, 97.5-102.5% (AT)		Sigma Aldrich	Cat#89803	
Pyridine		Sigma Aldrich	Cat#360570	
N,O-bis(trimetylsilyl)trifluoroacetamide and 1% trimethylchlorosilane		Sigma Aldrich	Cat#15238	
Critical commercial assays				
NAD/NADH Quantitation Kit		Sigma Aldrich	Cat#MAK037	
NADP/NADPH Quantitation Kit		Sigma Aldrich	Cat#MAK038	
ADP/ATP Ratio Assay Kit		Sigma Aldrich	Cat#MAK-135	
Glucose (HK) Assay Kit		Sigma Aldrich	Cat#GAHK20	
Experimental models: Organisms/strains				
*S. pombe nde1* Δ*::kanR h?*		This paper	SO8547	
*S. pombe ndi1* Δ*::kanR h-*		This paper	SO8587	
*S. pombe rip1* Δ*::kanR h-*		This paper	SO8688	
*S. pombe sdh3*Δ*::kanR h-*		This paper	SO8695	
*S. pombe cox6*⊿*::kanR h-*		This paper	SO8727	
*S. pombe gpd1*Δ*::hygR h?*		This paper	SO8991	
*S. pombe gpd1*Δ*::hygR, ndi1*Δ*::kanR h?*		This paper	SO9034	
*S. pombe gpd1*Δ*::hygR, nde1* Δ*::kanR h?*		This paper	SO9036	
*S. pombe gpd1*Δ*::hygR, rip1*Δ*::kanR h?*		This paper	SO9038	
*S. pombe gpd1* Δ*::hygR, cox6*⊿*::kanR h?*		This paper	SO9040	
*S. pombe mdh1* Δ*::hygR h+*		This paper	SO9046	
*S. pombe fum1* Δ*::kanR h-*		This paper	SO9078	
*S. pombe pyk1T343A h-*		Kamrad et al.^63^	SO9109	
*S. pombe pyk1T343A, cox6*⊿*::kanR h-*		This paper	SO9271	
*S. japonicus gpd1* Δ*::natMX6 h+*		This paper	SOJ3553	
*S. japonicus var. versatilis sjk4 h90 iodine stain positive*		Yu et al.^44^ and Klar^45^		SOJ3571	
*S. japonicus Wild isolate 1; YH156 from* *oak bark, Northeastern Pennsylvania, U.S.A.*		Osburn et al.^43^	SOJ3572	
*S. japonicus Wild isolate 2; YH157 from* *oak bark, Northeastern Pennsylvania, U.S.A.*		Osburn et al.^43^	SOJ3573	
*S. japonicus Wild isolate 3; from Matsue, Japan*		Kaino et al.^36^	SOJ3574	
*S. japonicus Wild isolate 4; from Hirosaki, Japan*		Kaino et al.^36^	SOJ3575	
*S. japonicus Wild isolate 5; from Nagano, Japan*		Kaino et al.^36^	SOJ3576	
*S. japonicus mdh1* Δ*::hygR h-*		This paper	SOJ3884	
*S. japonicus pyk1A343T h-*		This paper	SOJ3910	
*S. japonicus ndi1* Δ*::kanR h-*		This paper	SOJ4540	
*S. japonicus rip1* Δ*::kanR h-*	This paper			SOJ4542
*S. japonicus nde1* Δ*::kanR h-*	This paper			SOJ4543
*S. japonicus cox6*⊿*::kanR h-*	This paper			SOJ4545
*S. japonicus sdh3*Δ*::kanR h-*	This paper			SOJ5182
*S. japonicus ndi1* Δ*::kanR h+*	This paper			SOJ5199
*S. japonicus nde1* Δ*::kanR h+*	This paper			SOJ5201
*S. japonicus rip1* Δ*::kanR h+*	This paper			SOJ5203
*S. japonicus cox6*⊿*::kanR h+*	This paper			SOJ5204
*S. japonicus fum1* Δ*::kanR h-*	This paper			SOJ5212
Software and algorithms				
MANIC	Behrends et al.^92^			N/A
Masshunter Workstation Qualitative	Agilent Technologies			N/A
Analysis 10.0				
ImageJ	Schindelin et al.^93^			N/A
Growthcurver	Sprouffske and Wagner ^94^			N/A
Other				
Hanna waterproof field Dissolved	Scientific Laboratory Supplies			Cat#PHM0358D2
Oxygen meter with BOD				
VICTOR Nivo Multimode Plate Reader	Perkin Elmer			N/A

## Resource Availability

### Lead contact

Further information and requests for resources and reagents should be directed to and will be fulfilled by the Lead Contact, Snezhana Oliferenko (snezhka.oliferenko@crick.ac.uk).

## Experimental Model And Subject Details

*S. pombe* and *S. japonicus* prototrophic strains used in this study are listed in key resources table. We used standard fission yeast media and methods.^95,96^ For non-fermentable conditions, we used EMM with 2% glycerol or 2% galactose and 0.1% glucose. The inclusion of 0.1% glucose is necessary for *S. pombe* growth in these conditions.^56,97^ Temperature-controlled 200 rpm shaking incubators we used for liquid cultures. For most experiments, yeasts were pre-cultured in Edinburgh Minimal Medium (EMM) at 30°C. Pre-cultures for growth, serial dilution or oxygen consumption experiments were grown in rich Yeast Extract and Supplements (YES) medium at 24°C. All cultures were grown in a 200rpm shaking incubator. The following day, cultures were diluted to an OD_595_ within lag-phase or early-exponential phase, as required, and allowed to grow to a desired OD_595_. Once cultures reached early (0.2-0.5 OD_595_) or mid-exponential phase (OD_595_ determined by each strain and condition’s OD_595_ at stationary phase), cells were collected.

In the case of growth experiments, post-dilution growth was tracked in a plate reader, as follows. Yeasts were pre-cultured in YES at 25°C until OD_595_ 0.1-0.6. Cultures were then washed in experimental medium and diluted to 0.1 OD_595_. Growth was measured every 10 min at 30°C using VICTOR Nivo multimode plate reader (PerkinElmer). Growth curves were plotted using Graphpad Prism and growth rates were calculated using the Growthcurver R package.^94^ All experiments were performed in three technical and at least three biological replicates. Technical replicates were three wells in a 96-well plate; biological replicates were independent growth experiments using freshly-defrosted batches of each strain.

Mating of *S. pombe* and *S. japonicus* strains was performed on SPA solid medium. Spores were dissected and germinated on YES agar plates.

## Method Details

### Serial dilution assays

Serial dilution assays were performed by preculturing cells in YES at 25°C overnight until early-exponential phase. Cultures were diluted to 2×10^6^ cells/ml and serially diluted by a factor of 10. 2ml of each dilution were inoculated on plates. Plates were typically incubated at 30°C for three days. All experiments were repeated three times, using freshly-defrosted strains.

### Hyphae formation assay

*S. japonicus* cultures grown at 24°C overnight in YES to exponential phase were centrifuged and washed once in YES. The equivalent of OD_595_ 0.5 was inoculated on Yeast Extract Glucose Malt Extract Agar plates and incubated at 30°C for five days. Surface colonies were gently washed away to retain only the hyphae formed within the agar. Plates were imaged and ImageJ^93^ was used to measure the diameter of hyphal zone. Experiments were repeated at least thrice using freshly defrosted strains.

### Sporulation efficiency assay

*S. japonicus* wild-type and deletion strains of the opposite mating types, carrying the KanMX (KanR) selection marker, were crossed on SPA plates overnight at 30°C. The following day, a defined numbers of spores were dissected on YES plates using a Singer MSM micromanipulator (Singer Instruments). Between 50 and 100 spores per biological replicate were dissected. Spores were allowed to germinate over three days at 30°C. Spores that formed colonies versus the total spores dissected were counted to estimate sporulation efficiency. Colonies of spores from wild-type and deletion strain crosses were replica-plated onto YES plates with or without G418 sulphate (Sigma Aldrich) and spores that grew in the presence of G418 versus normal YES plates were counted to determine the KanMX positivity score. Experiments were replicated at least three times.

### Molecular genetics

Molecular genetic manipulations were performed via homologous recombination using the gene deletion cassette method,^98,99^ where target genes’ open reading frames were replaced by kanR, NatR or HygR cassettes flanked by 80-base-pair portions of the 5’ and 3’ UTRs of the gene of interest.

*S. pombe* was transformed using the lithium acetate method, as previously described.^100^ Briefly, early-exponential *S. pombe* cultures grown in YES were centrifuged and washed twice in dH_2_O. Cells were then washed in lithium acetate Tris-EDTA and incubated in 100ml of the same buffer for 10 min at room temperature together with 5mg of linear DNA and 50mg of sonicated salmon sperm DNA (Agilent Technologies). 240Δl of PEG-lithium acetate Tris-EDTA was added and cell suspension was mixed by swirling with a pipette tip. Samples were incubated at 30°C for 30–60 min. 43Δl of DMSO was then added and cells were washed in dH_2_O twice. Cells were then recovered in 10ml of YES at 25°C overnight and subsequently inoculated on selective plates – YES plates containing 100mg/mL G418 sulphate (Sigma Aldrich), 50Δg/mL hygromycin B (Sigma Aldrich), or 100Δg/mL nourseothricin (Werner BioAgents, Germany).

*S. japonicus* was transformed using electroporation.^96^ Briefly, early-exponential cultures grown in YES were pelleted and from then on kept on ice. Cells were washed three times using ice-cold dH_2_O and then were suspended in 5ml of cold 1M sorbitol with 50mM dithiothreitol in dH_2_O. Suspensions were incubated for 12 min at 30°C without shaking, after which cells were centrifuged and washed twice with cold 1M sorbitol. Pellets were then resuspended in 100Δl 1M sorbitol containing linearised DNA and sonicated salmon sperm DNA (Agilent Technologies). Cells were incubated on ice for 30 min. *S. japonicus* was subsequently electroporated at 2.30keV using a cold 2mm Gene Pulser/MicroPulser Electroporation Cuvette (Bio-Rad laboratories). Immediately after electroporation, 1ml of cold 1M sorbitol was added to the cell suspension, and cells were recovered overnight at 25°C in 10ml YES, shaking. The next day, cells were plated on selective plates, as with *S. pombe*.

### Oxygen consumption

Oxygen levels were measured using a waterproof field Dissolved Oxygen meter (Hanna HI 98193). Oxygen consumption was measured in mid-exponential cultures grown in YES. Cultures were centrifuged, cells were resuspended in fresh medium and used to fill a conical flask to the brim. The probe was submerged into the culture and the flask was sealed. Cultures were kept in gentle motion using a magnetic stirrer. Once oxygen readings stabilised, oxygen levels were recorded every minute for 10 min, after which the OD_595_ of cultures were recorded. Cultures were at room temperature during oxygen readings. Oxygen concentration over the time course was plotted to identify when oxygen changes slowed or plateaued. Selected linear oxygen changes were used to calculate the oxygen consumption rate per minute, which were normalised to the culture OD_595_. Measurements were independently repeated three times to yield three biological replicates.

### Biomass yield coefficient determination

Cells were pre-cultured in YES until early-exponential phase, after which they were diluted in EMM with 2% glucose to OD_595_ 0.1 and incubated at 30°C until stationary phase was reached. 5ml of cultures were dried over 48 h at 70°C and pellets were weighed. Alongside the dry weight, we measured glucose levels in EMM and the conditioned EMM at the time of collection using Glucose (HK) Assay Kit (Sigma Aldrich) as per the manufacturer’s instructions. The biomass yield coefficient was calculated using the dry weight divided by the change in glucose levels over the growth period. Data shown is the result of three technical and two biological replicates.

### Glucose consumption

Cells were pre-cultured in EMM with 2% glucose and once cultures reached early exponential phase, they were diluted to OD_595_ 0.1 and incubated overnight at 30°C. When cultures reached mid-exponential phase, cells were centrifuged and resuspended in fresh medium. Cultures were placed in a shaking incubator at 30°C and media samples were collected after 2 h. The percentage of glucose consumed during the incubation time was normalised to the change in OD_595_ in the same period, and the result was multiplied by the growth rate of each strain (h^-1^). Glucose was quantified using Glucose (HK) Assay Kit (Sigma Aldrich) as per the manufacturer’s instructions. Samples were collected as independent biological replicates from cultures grown on separate occasions, using freshly-defrosted strains.

### NAD(P)+/NAD(P)H quantification

Cells were pre-cultured in EMM overnight at 30°C until early-exponential phase. Subsequently, cultures were diluted to OD_595_ 0.1 and were grown overnight at 30°C. The equivalent of OD_595_ 5 of early-exponential (in the case of NAD^+^/NADH) or mid-exponential (in the case of NADP^+^/NADPH) cultures were harvested by centrifugation and snap-frozen in liquid nitrogen. NAD^+^/NADH was extracted and measured using MAK037 (Sigma Aldrich) and NADP^+^/NADPH was extracted and measured using MAK038 (Sigma Aldrich) as per manufacturer’s instructions. Cells were resuspended in chilled extraction buffer and lysed using lysing matrix Y tubes (MP Biomedicals) containing 0.5 mm diameter yttria-stabilized zirconium oxide beads and a cell disruptor (MP Biomedicals). Samples were kept as cold as possible by bead beating in 10 second intervals, at 6.5m/sec, 10 times, with 2-min breaks on ice between each round. Samples were then filtered through a 10kDa protein filter (MRCPRT010, Sigma Aldrich) via centrifugation at 4°C. Extracts were then processed as per the manufacturer’s instructions. Samples were collected in at least three independent experiments. Quantification was performed using a Tecan Spark plate reader.

### ATP/ADP quantification

Cells were pre-cultured in EMM overnight at 30°C until early-exponential phase. Subsequently, cultures were diluted to OD_595_ 0.1 and were grown overnight at 30°C. Early-exponential cultures in EMM were collected by quenching the equivalent of OD_595_ 5 cells in -80°C methanol. Suspensions were centrifuged at 3000rpm for 2 min at 4°C and decanted, and pellets were dried in -80°C overnight. ATP and ADP were extracted and quantified using the ATP/ADP ratio quantification kit (MAK-135, Sigma Aldrich) as per the manufacturer’s instructions, with the following modification. To extract ATP and ADP, cell pellets were lysed in the kit’s assay buffer using lysing matrix Y tubes (MP Biomedicals) containing 0.5 mm diameter yttria-stabilized zirconium oxide beads and a cell disruptor. Samples were kept as cold as possible by bead beating once for 10 seconds at 6.5m/sec at 4°C. Bioluminescence was quantified using a Tecan Spark microplate reader. Samples were collected as at least three biological replicates from cultures grown on separate occasions, using freshly defrosted strains.

### Gas chromatography-mass spectrometry metabolomics

For metabolomics experiments, cells were pre-cultured in EMM and diluted the previous day so that cells were in early-exponential phase at the time of harvest. In the case of stable isotope tracing experiments, pre-cultures and experimental cultures were both grown in EMM at 24°C. When cultures reached an OD_595_ of 0.2-0.4, cells were centrifuged at 3000rpm for 2 min and resuspended in either unlabelled (^12^C) or labelled (^13^C) media without dilution. Labelled media refers to EMM with 2% D-Glucose (U-^13^C_6_) (Cambridge Isotope Laboratories, Inc.). Once cells came into contact with labelled media, a time course was started. Cells were kept agitated until each time point was reached. At 1, 3, 5, 10, 30 or 60 min, a volume equivalent to 1.5 OD_595_ was injected into 100% LCMS-grade methanol (Sigma Aldrich) pre-cooled to -80°C. Quenched cultures were centrifuged and washed twice with -80°C methanol, centrifuging at 4°C and 3000rpm. The dried pellets stored at -80°C until extraction. For abundance quantification, a total of six replicates were collected per condition, two technical over three biological repeats. For stable isotope tracing, a total of four replicates were collected, two biological repeats with two technical replicates. Biological replicates were defined as independent experiments performed using freshly defrosted batches of cells.

The extraction protocol is a modified version of a method developed in Vowinckel et al.^23^ and Doppler et al.^101^ Cell pellets were resuspended in 200μl of LCMS-grade acetonitrile/methanol/water (2:2:1) (Sigma Aldrich) chilled to -20°C and transferred to lysing matrix Y tubes (MP Biomedicals) containing 0.5 mm diameter yttria-stabilized zirconium oxide beads. Extraction blanks were included from this stage, consisting of 200μl of extraction solution. 1nmol of scyllo-inositol (Sigma Aldrich) standard was added to all samples at this stage. Samples were kept on ice and lysed at 4°C. Bead beating was performed at 6.5m/sec for 10 seconds, five times, with 2-min breaks on ice after each round. Subsequently, samples were centrifuged at 12,000rpm for 2 min at 4°C and supernatants were dried for 1–2 h in a SpeedVac Vacuum Concentrator at 30°C. Samples were then stored at -80°C.

Dried extracts were resuspended in -20°C chilled 50μl LCMS-grade chloroform (Sigma Aldrich) and 300ml LCMS-grade methanol:-water (1:1) (Sigma Aldrich). Samples were vortexed for 1 min and centrifuged at 12,000rpm for 5 min at 4°C. 240μl of upper, polar phase was transferred to GC-MS glass vial inserts for drying, which included two 30μl methanol washes to ensure there was no residual water.

Derivatisation was performed based on published work.^51^ Samples were resuspended in 20μl of 20mg/ml of freshly dissolved methoxyamine hydrochloride (Sigma Aldrich) in pyridine (Sigma Aldrich). Samples were briefly vortexed and centrifuged, and incubated at room temperature overnight (around 15 h). The next day, 20μl of room-temperature N,O-bis(trimetylsilyl)trifluoroacetamide and 1% trimethylchlorosilane (Sigma Aldrich) was added to each sample, followed by a brief vortex and centrifugation.

Metabolites were detected using Agilent 7890B-MS7000C GC-MS as previously described.^51^ Samples were arranged and processed in random order together with regular hexane washes and metabolite standards (kindly gifted by James I. MacRae, Francis Crick Institute). Splitless injection was performed at 270°C in a 30m + 10m x 0.25mm DB-5MS+DG column (Agilent J&W). Helium was used as the carrier gas. The oven temperature cycle was as follows: 70°C for 2 min, temperature gradient up to 295°C with a rate of 12.5°C/min and gradient up to 350°C at a rate of 25°C/min. The 350°C temperature was held for 3 min. Electron impact ionization mode was used for MS analysis.

### Analysis of gas chromatography-mass spectrometry metabolomics data

Samples were analysed using a combination of MassHunter Workstation (Agilent Technologies) and MANIC, an updated version of the software GAVIN,^92^ for identification and integration of defined ion fragment peaks. For abundance quantification, integrals, the known amount of scyllo-inositol internal standard (1nmol) and the known abundances in the standardised metabolite mix (kindly gifted by James I. Macrae, Francis Crick Institute) run in parallel to samples were used to calculate an estimated nmol abundance of each metabolite in each sample. When presenting secreted metabolites, abundances of metabolites of interest and glucose were corrected to levels detected in the unconsumed EMM, and the change in levels of metabolites of interest were divided by the concomitant decrease in glucose. The formula used for calculating molar abundances as shown in Equation 1.

^13^C-glucose stable isotope tracing metabolomics data was analysed using MANIC to extract mass isotopologue ratios of metabolites of interest and percentage of metabolite pool that was labelled with ^13^C. Glycerol-3-phosphate synthesis in a defined time period was obtained by first identifying the timepoint when the proportion of ^13^C-labelling increased linearly (1 min), and normalising fractional labelling by the abundance of the total metabolite pool, to quantify the nmol of ^13^C metabolite generated after a specific time passed since exposure to ^13^C-glucose. Note that mass isotopologue ratios were obtained before steady state to capture transient isotopologues.

Data generated in metabolomics experiments performed for this study are shown in Data S1. Table S1 lists the nmol abundances of metabolites analysed in the standardised metabolite mix run in parallel to samples in each metabolomics experiment.

(1) $$\begin{array}{c} \;MRFF\;=\frac{SI\left[{\text{nmol}}_{\text{mm}}\right]}{met\left[{\text{nmol}}_{\text{mm}}\right]}\times \frac{met\left[{\text{int}}_{\text{mm}}\right]}{\text{SI}\left[{\text{int}}_{\text{mm}}\right]}\\ met\left[{\text{nmol}}_{\text{S}}\right]=\frac{\left[\frac{\text{met}\;int\text{S}}{\text{Sl}\;int\text{S}}\right]}{\text{MRFF}} \end{array}$$

Equation 1 - Formula for nmol abundance quantification

MRRF = molar relative response factor; SI = scyllo-inositol (internal standard); met = metabolite to be quantified; mm = standard metabolite mix; int = integrals; s = samples.

## Statistical Analyses

The statistical details of experiments, including the number of biological and technical replicates and the dispersion and precision measures can be found in figure legends and methods details. All data were analysed using unpaired t-test statistical analysis, unless indicated otherwise. All plots were generated using Graphpad Prism.

## Supplementary Material

Supplementary Information

## Data Availability

All unique reagents generated in this study are available from the Lead Contact without restriction.

## Highlights

- *S. japonicus* relies on dihydroxyacetone phosphate reduction to oxidize NADH
- Fermenting fission yeasts operate a bifurcated TCA pathway to produce biomass
- *S. japonicus* optimizes glycolysis to generate high ATP levels without respiration

## Data and code availability

- Metabolomics raw data is supplied in Data S1. Other types of raw data will be shared by the lead contact upon request.
- This paper does not report original code.
- Any additional information required to reanalyse the data reported in this paper is available from the lead contact upon request.
