# Supplementary material for: Optimization of energy production and central carbon metabolism in a non-respiring eukaryote
Source: Curr Biol. Author manuscript; Available in PMC 2024 Feb 16. (PMC7615655; doi:10.1016/j.cub.2023.04.046)
Supplement: Supplementary Information [file EMS193942-supplement-Supplemental_Information.pdf]

**Current Biology, Volume *33***

## Supplemental Information

# Optimization of energy production and central carbon metabolism in a non-respiring eukaryote

**Sara Alam, Ying Gu, Polina Reichert, Jürg Bähler, and Snezhana Oliferenko**

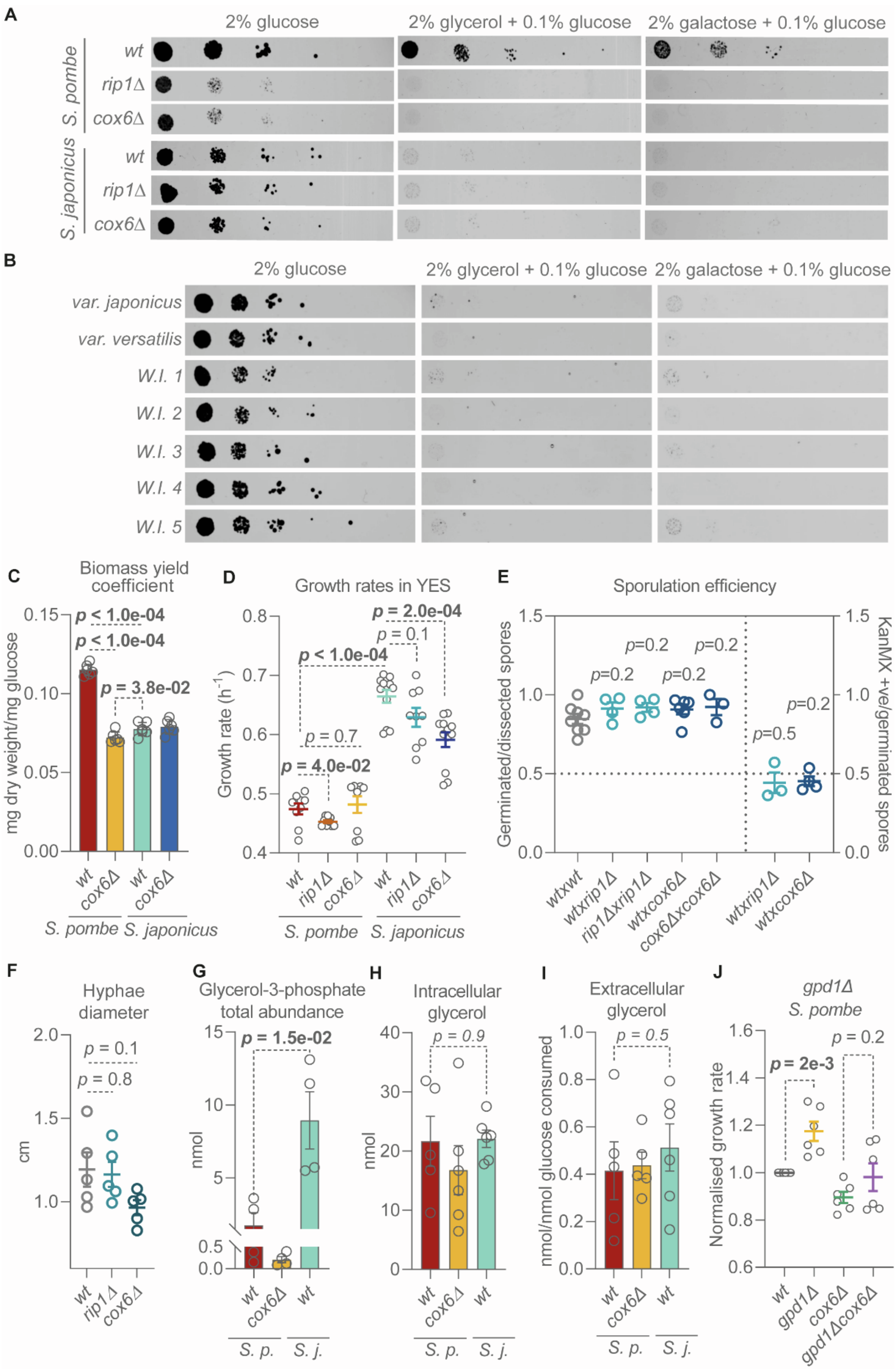
A
2% glucose
2% glycerol + 0.1% glucose
2% galactose + 0.1% glucose
S. pombe
S. japonicus
wt
rip1Δ
cox6Δ
B
2% glucose
2% glycerol + 0.1% glucose
2% galactose + 0.1% glucose
var. japonicus
var. versatilis
W.I. 1
W.I. 2
W.I. 3
W.I. 4
W.I. 5
C
Biomass yield coefficient
mg dry weight/mg glucose
p < 1.0e-04
p < 1.0e-04
p = 3.8e-02
S. pombe
S. japonicus
D
Growth rates in YES
Growth rate (h-1)
p = 2.0e-04
p = 0.1
p < 1.0e-04
p = 0.7
p = 4.0e-02
S. pombe
S. japonicus
E
Sporulation efficiency
Germinated/dissected spores
KanMX +ve/germinated spores
p=0.2
p=0.2
p=0.2
p=0.2
p=0.2
p=0.5
wtxwt
wtxrip1Δ
rip1Δxrip1Δ
wtxcox6Δ
cox6Δxcox6Δ
wtxrip1Δ
wtxcox6Δ
F
Hyphae diameter
cm
p = 0.1
p = 0.8
G
Glycerol-3-phosphate total abundance
nmol
p = 1.5e-02
S. p.
S. j.
H
Intracellular glycerol
nmol
p = 0.9
S. p.
S. j.
I
Extracellular glycerol
nmol/nmol glucose consumed
p = 0.5
S. p.
S. j.
J
gpd1Δ
S. pombe
Normalised growth rate
p = 2e-3
p = 0.2
wt
gpd1Δ
cox6Δ
gpd1Δcox6Δ

**Figure S1. *S. japonicus* does not respire and does not generate more glycerol than *S. pombe*, Related to Figure 1 and Data S1B, C.**

(**A**) Serial dilution assays showing growth of *S. pombe* and *S. japonicus* of indicated genotypes on EMM agar with either 2% (w/v) glucose, 2% (v/v) glycerol or 2% (w/v) galactose with 0.1% (w/v) glucose. Serially diluted strains were grown over four days at 30˚C, images are representative of three independent biological replicates. (**B**) Serial dilution assays showing growth of wild type *S. japonicus* strains on EMM, as in (**A**). *var japonicus* is our wild type lab strain. *var versatilis* is a lab strain from the USA. W.I, wild isolate; W.I.1: YH156 from oak bark, Northeastern Pennsylvania, U.S.A.; W.I.2: YH157 from oak bark, Northeastern Pennsylvania, U.S.A.; W.I.3-5: isolates from Matsue, Hirosaki and Nagano, Japan. (**C**) Biomass yield coefficient, in mg dry weight produced per mg of glucose consumed in EMM, of indicated *S. pombe* and *S. japonicus* strains. (**D**) Growth rates of indicated *S. pombe* and *S. japonicus* strains in YES medium. (**E**) *Left*, sporulation efficiency of *S. japonicus* crosses of indicated genotypes, calculated by counting the proportion of germinated spores to total dissected spores. *Right*, the recovery of KanMX-marked progeny calculated by counting spores that grew in the presence of G418 compared to total germinated spores. Spore germination score *p* values were generated using unpaired t-test, KanMX positivity score *p* values were calculated using one sample t-test assuming a Gaussian distribution, testing against a hypothetical mean of 0.5. Plots represent means ±SEM of two technical and two or three biological replicates. (**F**) Hyphal zone diameters measured five days after inoculation on yeast extract, glucose and malt extract agar plates. (**G**) Intracellular glycerol-3-phosphate abundance in exponentially growing *S. pombe* and *S. japonicus* in EMM. (**H**) as (**G**) but for glycerol. (**I**) Glycerol detected in the conditioned EMM medium of *S. pombe* and *S. japonicus* cultures, normalised to the levels of glucose consumed. (**G-I**) Raw data can be found in Data S1B and S1C. (**J**) Growth rates of *S. pombe* deletion strains in EMM. (**C, D, F-J**) Plots represent means ±SEM of at least two biological and two to three technical replicates. *p* values were generated using unpaired t-test.

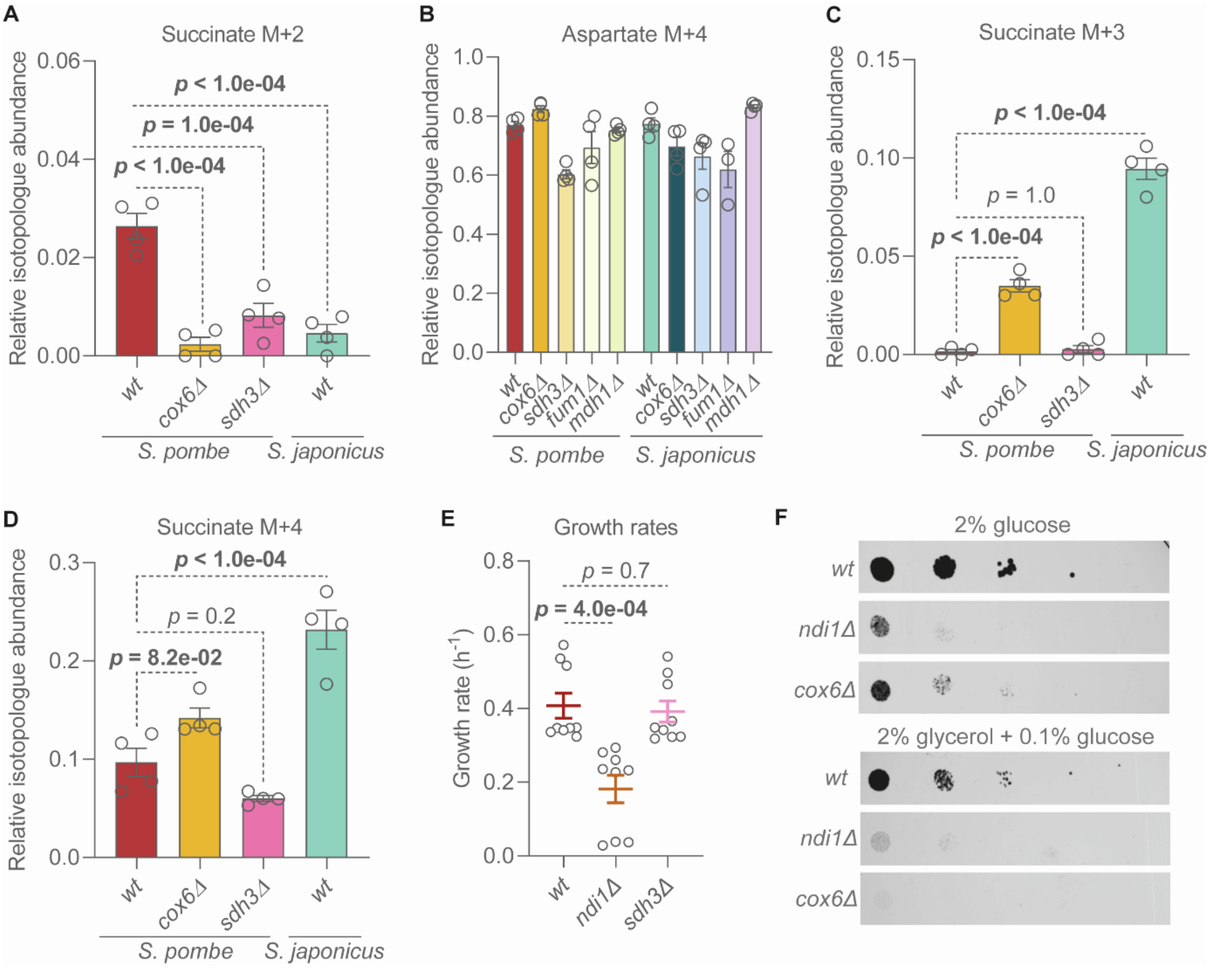


**Figure S2. Fermenting *S. pombe* and *S. japonicus* operate a bifurcated TCA pathway, Related to Figure 2 and Data S1D.**

(**A**) Proportion of M+2 succinate relative to the entire pool of detected succinate in *S. pombe* and *S. japonicus* 30 minutes after $^{13}C_6$ glucose addition. (**B**) Proportion of M+4 aspartate (proxy for oxaloacetate) relative to the detected pool of aspartate in *S. pombe* and *S. japonicus* 30 minutes after $^{13}C_6$ glucose addition. (**C**, **D**) Proportion of M+3 and M+4 succinate relative to the entire pool of detected succinate in *S. pombe* and *S. japonicus* 30 minutes after $^{13}C_6$ glucose addition. (**A-D**) Shown are means ±SEM of two biological and two technical replicates, p values calculated using unpaired t-test. Raw data can be found in Data S1D. (**E**) Growth rates of indicated *S. pombe* strains in EMM. Shown are means ±SEM of three biological and three technical replicates, p values calculated using unpaired t-test. (**F**) Serial dilution assays of *S. pombe* on EMM agar plates with either 2% (w/v) glucose or 2% (v/v) glycerol with 0.1% (w/v) glucose. Serially diluted strains were grown over four days at 30˚C, images are representative of three independent biological replicates.

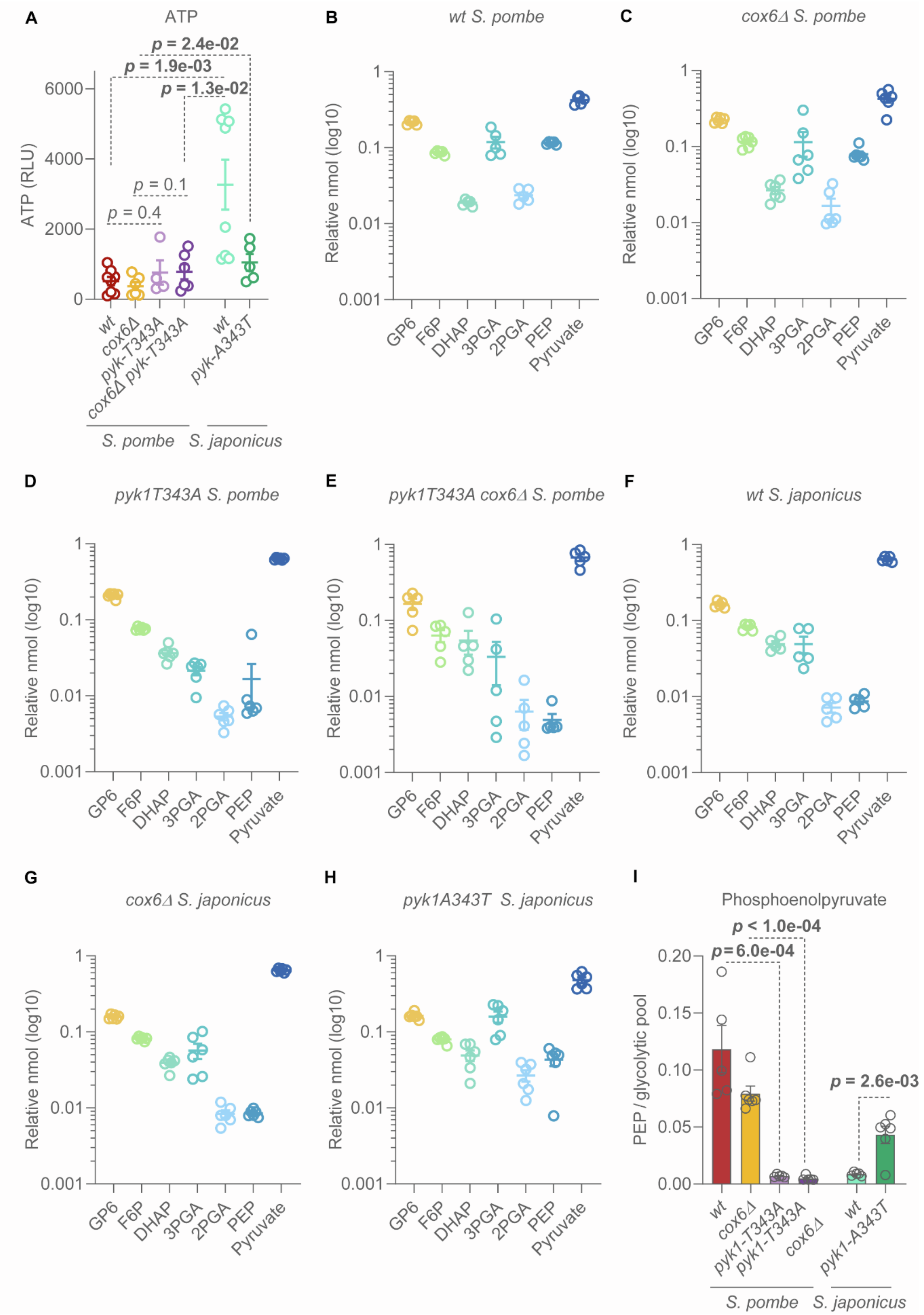

A
ATP
p = 2.4e-02
p = 1.9e-03
p = 1.3e-02
p = 0.1
p = 0.4
ATP (RLU)
6000
4000
2000
0
wt
cox6Δ
pyk-T343A
cox6Δ pyk-T343A
wt
pyk-A343T
S. pombe
S. japonicus
B
wt S. pombe
C
cox6Δ S. pombe
D
pyk1T343A S. pombe
E
pyk1T343A cox6Δ S. pombe
F
wt S. japonicus
G
cox6Δ S. japonicus
H
pyk1A343T S. japonicus
Relative nmol (log10)
1
0.1
0.01
0.001
GP6
F6P
DHAP
3PGA
2PGA
PEP
Pyruvate
I
Phosphoenolpyruvate
p < 1.0e-04
p = 6.0e-04
p = 2.6e-03
PEP / glycolytic pool
0.20
0.15
0.10
0.05
0.00
wt
cox6Δ
pyk1-T343A
pyk1-T343A cox6Δ
wt
pyk1-A343T
S. pombe
S. japonicus

**Figure S3. *S. japonicus* has a higher glycolytic activity as compared to *S. pombe*, Related to Figure 3 and Data S1E**

(**A**) ATP levels quantified by relative light units (RLU) in a luciferase-based ATP assay of whole-cell extracts. Shown are means ±SEM values of at least four biological replicates. *p* values were calculated using unpaired t-test. (**B-H**) Relative abundances of glycolytic intermediates in *S. pombe* and *S. japonicus*. Detected glycolytic intermediates were quantified and the relative proportion of each metabolite relative to the sum of the abundances of G6P, F6P, DHAP, 3PGA, 2PGA, PEP and pyruvate were plotted. (**I**) Phosphoenolpyruvate abundance relative to the sum of detected glycolytic intermediates (G6P, F6P, DHAP, 3PGA, 2PGA, PEP and pyruvate). (**B-I**) Plotted values are means ±SEM of three technical and two biological replicate experiments. *p* values were calculated using unpaired t-test. Raw data can be found in Data S1E.

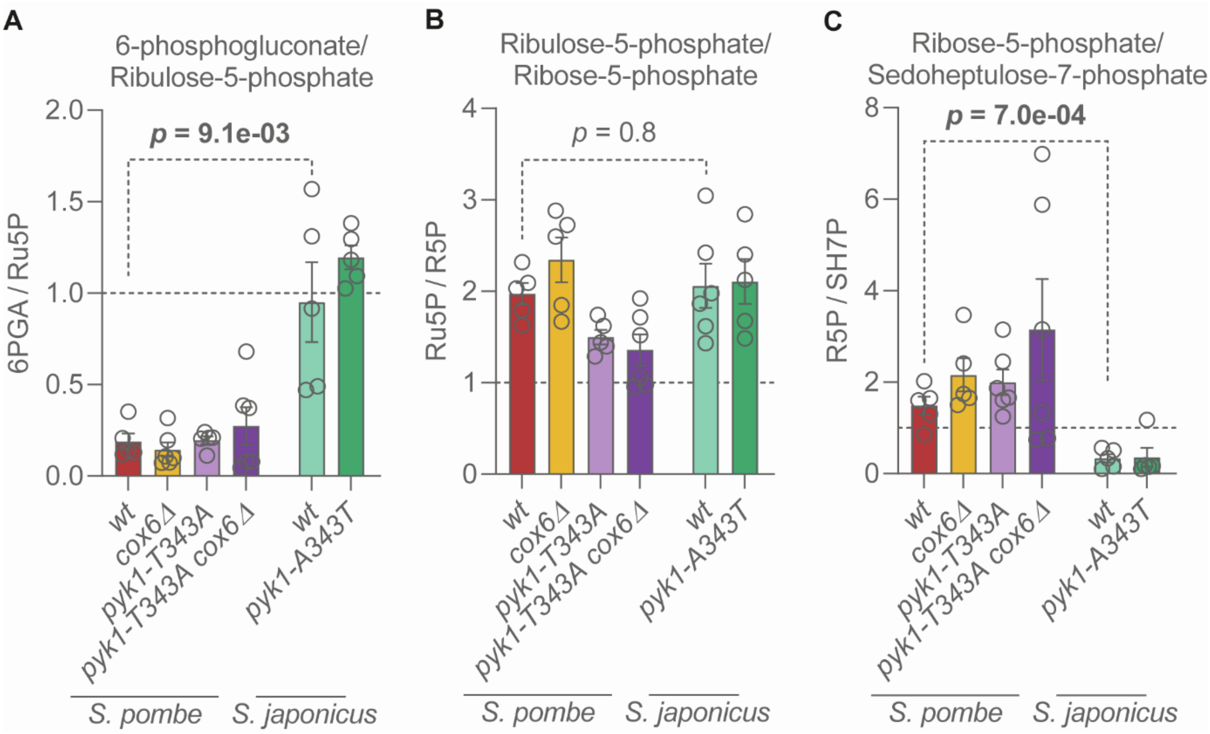


**Figure S4. The pentose phosphate pathway exhibits distinct regulatory points in *S. pombe* and *S. japonicus*, Related to Figure 4 and Data S1E.**

(**A**) Abundance of 6-phosphogluconate normalised to ribulose-5-phosphate. (**B**) Abundance of ribulose-5-phosphate normalised to that of ribose-5-phosphate. Ribulose-5-phosphate appears to be more abundant than ribose-5-phosphate, which may indicate a slow entry into the non-oxidative PPP in both *S. pombe* and *S. japonicus*. (**C**) Ribose-5-phosphate abundance normalised to sedoheptulose-7-phosphate. This ratio is below 1 in *S. japonicus* which indicates high levels of sedoheptulose-7-phosphate. Sedoheptulose-7-phosphate synthesis also produces fructose-6-phosphate, capable of re-entry into glycolysis. (**A-C**) Plotted are the means ±SEM of two biological and three technical replicates. Dotted line indicates the ratio of 1. Statistical analyses were performed using unpaired t-tests. Raw data can be found in Data S1E.

| Metabolite | Abundance in standard metabolite mix (nmol) |
|---|---|
| 2-phosphoglycerate | 5 |
| 3-phosphoglycerate | 5 |
| 6-phosphogluconate | 5 |
| Alanine | 5 |
| alpha-ketoglutarate | 10 |
| Arabinose | 0.5 |
| Aspartate | 5 |
| Citrate | 2.5 |
| Dihydroxyacetone phosphate | 5 |
| Fructose-6-phosphate | 2.5 |
| Fumarate | 2.5 |
| Glucose | 0.5 |
| Glucose-6-phosphate | 2.5 |
| Glutamate | 5 |
| Glycerol | 5 |
| Glycerol-3-phosphate | 1 |
| Glycine | 5 |
| Lactate | 2.5 |
| Malate | 2.5 |
| Phenylalanine | 5 |
| Phosphoenolpyruvate | 5 |
| Pyruvate | 20 |
| Ribose | 0.5 |
| Ribose-5-phosphate | 2.5 |
| Ribulose-5-phosphate | 2.5 |
| Scyllo-inositol | 0.5 |
| Sedoheptulose-7-phosphate | 10 |
| Succinate | 2.5 |
| Tyrosine | 5 |
| Uracil | 5 |

**Table S1. Composition of standard metabolite mix, Related to STAR Methods and Data S1.**

Composition of standard metabolite mix run alongside samples in GC-MS metabolomics experiments to assist with identification and quantification of metabolites of interest. Standard metabolite mix was kindly gifted by James I. MacRae, Francis Crick Institute.
